# 5-Bromo-2-(2-fluoro­phen­yl)-3-methyl­sulfinyl-1-benzo­furan

**DOI:** 10.1107/S1600536813010519

**Published:** 2013-04-24

**Authors:** Hong Dae Choi, Pil Ja Seo, Uk Lee

**Affiliations:** a Department of Chemistry, Dongeui University, San 24 Kaya-dong, Busanjin-gu, Busan 614-714, Republic of Korea; b Department of Chemistry, Pukyong National University, 599-1 Daeyeon 3-dong, Nam-gu, Busan 608-737, Republic of Korea

## Abstract

In the title compound, C_15_H_10_BrFO_2_S, the 2-fluoro­phenyl ring makes a dihedral angle of 32.28 (6)° with the mean plane [r.m.s. deviation = 0.010 (1) Å] of the benzo­furan fragment. In the crystal, mol­ecules are linked by weak C—H⋯O hydrogen bonds and Br⋯O contacts [3.0917 (13) Å], forming a three-dimensional network.

## Related literature

For background information and the crystal structures of related compounds, see: Choi *et al.* (2010 ▶, 2012 ▶). For a review of halogen bonding, see: Politzer *et al.* (2007 ▶).
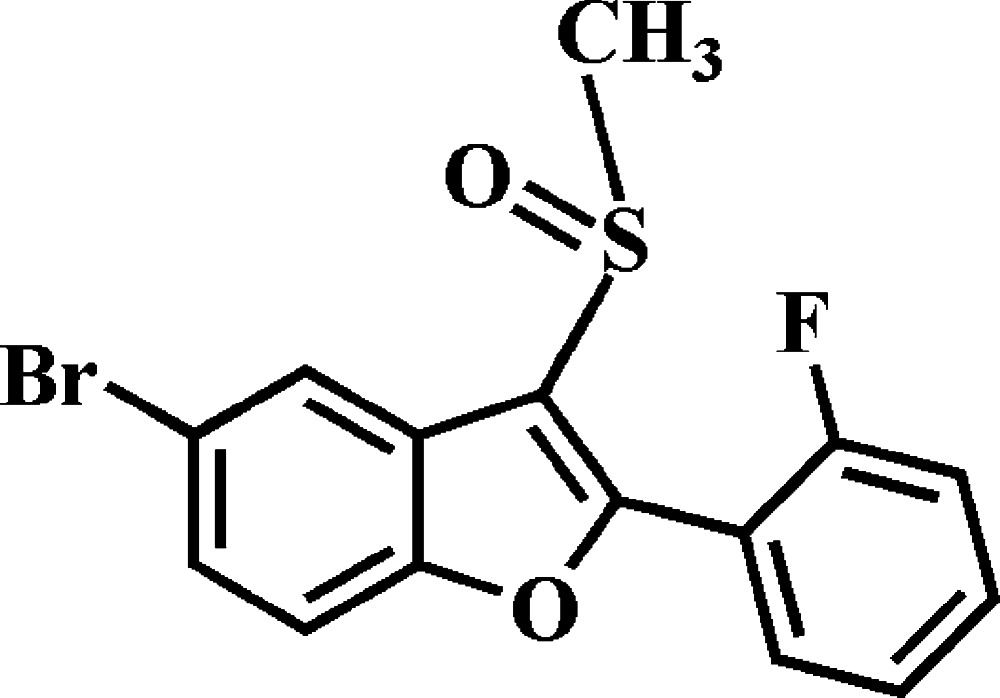


## Experimental

### 

#### Crystal data

C_15_H_10_BrFO_2_S
*M* *_r_* = 353.20
Triclinic, 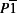

*a* = 7.9877 (1) Å
*b* = 8.3523 (2) Å
*c* = 10.8908 (2) Å
α = 93.146 (1)°
β = 94.605 (1)°
γ = 112.150 (1)°
*V* = 667.93 (2) Å^3^
*Z* = 2
Mo *K*α radiation
μ = 3.24 mm^−1^
*T* = 173 K
0.33 × 0.23 × 0.16 mm

#### Data collection

Bruker SMART APEXII CCD diffractometer
Absorption correction: multi-scan (*SADABS*; Bruker, 2009 ▶) *T*_min_ = 0.506, *T*_max_ = 0.746
12688 measured reflections
3333 independent reflections
3043 reflections with *I* > 2σ(*I*)
*R*_int_ = 0.037

#### Refinement

*R*[*F* ^2^ > 2σ(*F* ^2^)] = 0.026
*wR*(*F* ^2^) = 0.068
*S* = 1.07
3333 reflections
182 parameters
H-atom parameters constrained
Δρ_max_ = 0.54 e Å^−3^
Δρ_min_ = −0.47 e Å^−3^

### 

Data collection: *APEX2* (Bruker, 2009 ▶); cell refinement: *SAINT* (Bruker, 2009 ▶); data reduction: *SAINT*; program(s) used to solve structure: *SHELXS97* (Sheldrick, 2008 ▶); program(s) used to refine structure: *SHELXL97* (Sheldrick, 2008 ▶); molecular graphics: *ORTEP-3* (Farrugia, 2012 ▶) and *DIAMOND* (Brandenburg, 1998 ▶); software used to prepare material for publication: *SHELXL97*.

## Supplementary Material

Crystal structure: contains datablock(s) global, I. DOI: 10.1107/S1600536813010519/qk2058sup1.cif

Structure factors: contains datablock(s) I. DOI: 10.1107/S1600536813010519/qk2058Isup2.hkl

Supplementary material file. DOI: 10.1107/S1600536813010519/qk2058Isup3.cml

Additional supplementary materials:  crystallographic information; 3D view; checkCIF report

## Figures and Tables

**Table 1:** Hydrogen-bond geometry (Å, °)

*D*—H⋯*A*	*D*—H	H⋯*A*	*D*⋯*A*	*D*—H⋯*A*
C6—H6⋯O1^i^	0.95	2.52	3.4633 (19)	173
C14—H14⋯O2^ii^	0.95	2.44	3.365 (2)	164

Symmetry codes: (i) 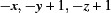; (ii) 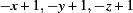.

**Fig. 1.**
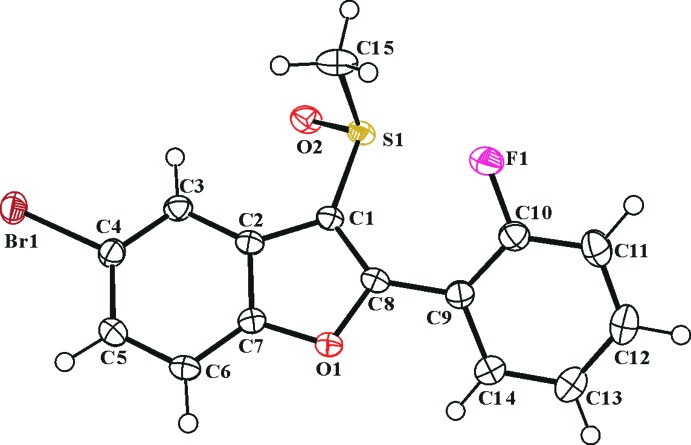
The molecular structure of the title compound with the atom numbering scheme. Displacement ellipsoids are drawn at the 50% probability level. H atoms are presented as small spheres of arbitrary radius.

**Fig. 2.**
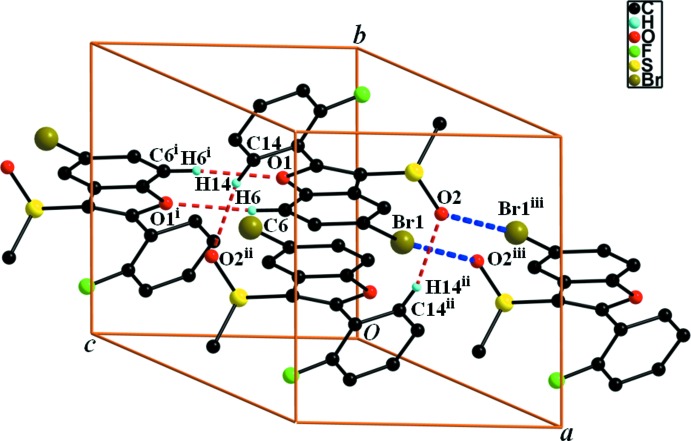
A view of the C–H···O and Br···O interactions (dotted lines) in the crystal structure of the title compound. H atoms non-participating in hydrogen-bonding were omitted for clarity. [Symmetry codes: (i) -x, -y+1, -z+1; (ii) -x+1, -y+1, -z+1; (iii) -x+1, -y+1, -z.]

## supplementary crystallographic information

### Comment

As part of our ongoing study of 5-bromo-3-methylsulfinyl-1-benzofuran derivatives containing 4-fluorophenyl (Choi *et al.*, 2010) and 3-fluorophenyl (Choi *et al.*, 2012) substituents in 2-position, we report herein the crystal structure of the title compound.

In the title molecule (Fig. 1), the benzofuran unit is essentially planar, with a mean deviation of 0.010 (1) Å from the least-squares plane defined by the nine constituent atoms. The dihedral angle between the 2-fluorophenyl ring and the mean plane of the benzofuran ring is 32.28 (6)°. In the crystal structure (Fig. 2), molecules are connected by weak C–H···O hydrogen bonds (Table 1) and by Br···O halogen-bondings between the bromine atom and the O atom of the S═O unit [Br1···O2(-*x*+1, -*y*+1, -*z*) = 3.0917 (13) Å, C4–Br1···O2(-*x*+1, -*y*+1, -*z*) = 170.46 (6)°] (Politzer *et al.*, 2007).

### Experimental

3-Chloroperoxybenzoic acid (77%, 202 mg, 0.9 mmol) was added in small portions to a stirred solution of 5-bromo-2-(2-fluorophenyl)-3-methylsulfanyl-1-benzofuran (270 mg, 0.8 mmol) in dichloromethane (30 mL) at 273 K. After being stirred at room temperature for 4h, the mixture was washed with saturated sodium bicarbonate solution and the organic layer was separated, dried over magnesium sulfate, filtered and concentrated at reduced pressure. The residue was purified by column chromatography (hexane/ethyl acetate, 2:1 v/v) to afford the title compound as a colorless solid [yield 72%, m.p. 454-456 K; *R*_f_ = 0.49 (hexane/ethyl acetate, 2:1 v/v)]. Single crystals suitable for X-ray diffraction were prepared by slow evaporation of a solution of the title compound in acetone at room temperature.

### Refinement

All H atoms were positioned geometrically and refined using a riding model, with C–H = 0.95 Å for aryl and 0.98 Å for methyl H atoms. *U*_iso_(H) = 1.2*U*_eq_(C) for aryl and 1.5*U*_eq_(C) for methyl H atoms. The positions of methyl hydrogens were optimized rotationally.

### Crystal data

**Table:**

C_15_H_10_BrFO_2_S	*Z* = 2
*M**_r_* = 353.20	*F*(000) = 352
Triclinic, *P*1	*D*_x_ = 1.756 Mg m^−^^3^
Hall symbol: -P 1	Melting point = 454–456 K
*a* = 7.9877 (1) Å	Mo *K*α radiation, λ = 0.71073 Å
*b* = 8.3523 (2) Å	Cell parameters from 6958 reflections
*c* = 10.8908 (2) Å	θ = 2.5–28.5°
α = 93.146 (1)°	µ = 3.24 mm^−^^1^
β = 94.605 (1)°	*T* = 173 K
γ = 112.150 (1)°	Block, colourless
*V* = 667.93 (2) Å^3^	0.33 × 0.23 × 0.16 mm

### Data collection

**Table:**

Bruker SMART APEXII CCD diffractometer	3333 independent reflections
Radiation source: rotating anode	3043 reflections with *I* > 2σ(*I*)
Graphite multilayer monochromator	*R*_int_ = 0.037
Detector resolution: 10.0 pixels mm^-1^	θ_max_ = 28.4°, θ_min_ = 1.9°
φ and ω scans	*h* = −10→10
Absorption correction: multi-scan (*SADABS*; Bruker, 2009)	*k* = −11→9
*T*_min_ = 0.506, *T*_max_ = 0.746	*l* = −14→14
12688 measured reflections	

### Refinement

**Table:**

Refinement on *F*^2^	Primary atom site location: structure-invariant direct methods
Least-squares matrix: full	Secondary atom site location: difference Fourier map
*R*[*F*^2^ > 2σ(*F*^2^)] = 0.026	Hydrogen site location: difference Fourier map
*wR*(*F*^2^) = 0.068	H-atom parameters constrained
*S* = 1.07	*w* = 1/[σ^2^(*F*_o_^2^) + (0.0343*P*)^2^ + 0.1931*P*] where *P* = (*F*_o_^2^ + 2*F*_c_^2^)/3
3333 reflections	(Δ/σ)_max_ < 0.001
182 parameters	Δρ_max_ = 0.54 e Å^−^^3^
0 restraints	Δρ_min_ = −0.47 e Å^−^^3^

### Special details

**Table:**

Geometry. All esds (except the esd in the dihedral angle between two l.s. planes) are estimated using the full covariance matrix. The cell esds are taken into account individually in the estimation of esds in distances, angles and torsion angles; correlations between esds in cell parameters are only used when they are defined by crystal symmetry. An approximate (isotropic) treatment of cell esds is used for estimating esds involving l.s. planes.
Refinement. Refinement of F^2^ against ALL reflections. The weighted R-factor wR and goodness of fit S are based on F^2^, conventional R-factors R are based on F, with F set to zero for negative F^2^. The threshold expression of F^2^ > 2sigma(F^2^) is used only for calculating R-factors(gt) etc. and is not relevant to the choice of reflections for refinement. R-factors based on F^2^ are statistically about twice as large as those based on F, and R- factors based on ALL data will be even larger.

### Fractional atomic coordinates and isotropic or equivalent isotropic displacement parameters (Å^2^)

**Table:**

	*x*	*y*	*z*	*U*_iso_*/*U*_eq_	
Br1	0.18802 (2)	0.38853 (2)	−0.025225 (15)	0.03036 (7)	
S1	0.79138 (5)	0.80682 (5)	0.39342 (4)	0.02209 (10)	
F1	0.76843 (15)	1.06406 (14)	0.57145 (10)	0.0342 (3)	
O1	0.30062 (15)	0.63538 (16)	0.51032 (10)	0.0230 (2)	
O2	0.81362 (17)	0.67220 (17)	0.30799 (12)	0.0288 (3)	
C2	0.4102 (2)	0.6196 (2)	0.32587 (15)	0.0204 (3)	
C3	0.3923 (2)	0.5656 (2)	0.20019 (15)	0.0226 (3)	
H3	0.4940	0.5985	0.1540	0.027*	
C4	0.2202 (2)	0.4623 (2)	0.14625 (15)	0.0233 (3)	
C5	0.0670 (2)	0.4087 (2)	0.21188 (17)	0.0259 (4)	
H5	−0.0484	0.3358	0.1707	0.031*	
C6	0.0840 (2)	0.4617 (2)	0.33594 (17)	0.0263 (4)	
H6	−0.0177	0.4280	0.3823	0.032*	
C7	0.2565 (2)	0.5663 (2)	0.38940 (15)	0.0215 (3)	
C8	0.4844 (2)	0.7334 (2)	0.52480 (15)	0.0211 (3)	
C9	0.5539 (2)	0.8209 (2)	0.64851 (16)	0.0220 (3)	
C1	0.5573 (2)	0.7274 (2)	0.41604 (15)	0.0204 (3)	
C10	0.6932 (2)	0.9831 (2)	0.66985 (16)	0.0254 (3)	
C11	0.7582 (3)	1.0711 (3)	0.78519 (18)	0.0318 (4)	
H11	0.8551	1.1819	0.7955	0.038*	
C12	0.6776 (3)	0.9923 (3)	0.88561 (18)	0.0351 (4)	
H12	0.7205	1.0491	0.9666	0.042*	
C13	0.5350 (3)	0.8319 (3)	0.86937 (17)	0.0323 (4)	
H13	0.4798	0.7804	0.9391	0.039*	
C14	0.4722 (2)	0.7457 (2)	0.75239 (16)	0.0253 (3)	
H14	0.3740	0.6358	0.7421	0.030*	
C15	0.7942 (3)	0.9770 (3)	0.2988 (2)	0.0347 (4)	
H15A	0.9165	1.0346	0.2740	0.052*	
H15B	0.7607	1.0617	0.3459	0.052*	
H15C	0.7070	0.9275	0.2249	0.052*	

### Atomic displacement parameters (Å^2^)

**Table:**

	*U*^11^	*U*^22^	*U*^33^	*U*^12^	*U*^13^	*U*^23^
Br1	0.02712 (10)	0.04006 (13)	0.02189 (10)	0.01183 (8)	0.00002 (7)	−0.00229 (7)
S1	0.01598 (18)	0.0224 (2)	0.0267 (2)	0.00551 (15)	0.00481 (15)	0.00222 (16)
F1	0.0343 (6)	0.0261 (6)	0.0343 (6)	0.0017 (4)	0.0110 (5)	0.0014 (5)
O1	0.0177 (5)	0.0262 (6)	0.0223 (6)	0.0049 (5)	0.0049 (4)	0.0011 (5)
O2	0.0247 (6)	0.0279 (7)	0.0348 (7)	0.0104 (5)	0.0099 (5)	−0.0007 (5)
C2	0.0178 (7)	0.0202 (8)	0.0238 (8)	0.0075 (6)	0.0043 (6)	0.0039 (6)
C3	0.0205 (7)	0.0259 (9)	0.0228 (8)	0.0096 (6)	0.0055 (6)	0.0035 (6)
C4	0.0243 (8)	0.0260 (9)	0.0201 (8)	0.0103 (7)	0.0022 (6)	0.0010 (6)
C5	0.0190 (7)	0.0285 (9)	0.0271 (9)	0.0062 (6)	0.0013 (6)	0.0011 (7)
C6	0.0181 (7)	0.0306 (9)	0.0277 (9)	0.0056 (7)	0.0070 (6)	0.0028 (7)
C7	0.0204 (7)	0.0232 (8)	0.0210 (8)	0.0079 (6)	0.0044 (6)	0.0025 (6)
C8	0.0169 (7)	0.0204 (8)	0.0252 (8)	0.0059 (6)	0.0035 (6)	0.0032 (6)
C9	0.0208 (7)	0.0236 (8)	0.0237 (8)	0.0107 (6)	0.0034 (6)	0.0013 (6)
C1	0.0173 (7)	0.0209 (8)	0.0224 (8)	0.0061 (6)	0.0040 (6)	0.0027 (6)
C10	0.0232 (8)	0.0265 (9)	0.0277 (9)	0.0105 (7)	0.0049 (6)	0.0013 (7)
C11	0.0297 (9)	0.0273 (10)	0.0361 (10)	0.0104 (7)	−0.0011 (7)	−0.0059 (8)
C12	0.0415 (11)	0.0387 (11)	0.0259 (9)	0.0187 (9)	−0.0023 (8)	−0.0062 (8)
C13	0.0398 (10)	0.0368 (11)	0.0241 (9)	0.0188 (8)	0.0045 (7)	0.0029 (7)
C14	0.0270 (8)	0.0264 (9)	0.0251 (8)	0.0125 (7)	0.0050 (7)	0.0045 (7)
C15	0.0338 (10)	0.0302 (10)	0.0430 (11)	0.0116 (8)	0.0163 (8)	0.0155 (8)

### Geometric parameters (Å, º)

**Table:**

Br1—C4	1.9001 (17)	C6—H6	0.9500
Br1—O2^i^	3.0917 (13)	C8—C1	1.368 (2)
S1—O2	1.4912 (13)	C8—C9	1.457 (2)
S1—C1	1.7756 (16)	C9—C10	1.383 (2)
S1—C15	1.795 (2)	C9—C14	1.410 (2)
F1—C10	1.358 (2)	C10—C11	1.376 (3)
O1—C7	1.371 (2)	C11—C12	1.384 (3)
O1—C8	1.3754 (19)	C11—H11	0.9500
C2—C7	1.394 (2)	C12—C13	1.384 (3)
C2—C3	1.396 (2)	C12—H12	0.9500
C2—C1	1.441 (2)	C13—C14	1.385 (3)
C3—C4	1.378 (2)	C13—H13	0.9500
C3—H3	0.9500	C14—H14	0.9500
C4—C5	1.404 (2)	C15—H15A	0.9800
C5—C6	1.378 (3)	C15—H15B	0.9800
C5—H5	0.9500	C15—H15C	0.9800
C6—C7	1.382 (2)		
			
C4—Br1—O2^i^	170.46 (6)	C10—C9—C8	122.76 (15)
O2—S1—C1	105.91 (8)	C14—C9—C8	120.24 (15)
O2—S1—C15	105.08 (9)	C8—C1—C2	106.87 (13)
C1—S1—C15	97.98 (8)	C8—C1—S1	127.23 (13)
C7—O1—C8	106.82 (12)	C2—C1—S1	125.23 (12)
C7—C2—C3	119.08 (15)	F1—C10—C11	117.24 (16)
C7—C2—C1	105.27 (14)	F1—C10—C9	118.65 (15)
C3—C2—C1	135.64 (14)	C11—C10—C9	124.09 (17)
C4—C3—C2	116.98 (14)	C10—C11—C12	117.63 (18)
C4—C3—H3	121.5	C10—C11—H11	121.2
C2—C3—H3	121.5	C12—C11—H11	121.2
C3—C4—C5	123.17 (16)	C13—C12—C11	120.72 (18)
C3—C4—Br1	118.57 (12)	C13—C12—H12	119.6
C5—C4—Br1	118.26 (13)	C11—C12—H12	119.6
C6—C5—C4	120.10 (16)	C12—C13—C14	120.58 (18)
C6—C5—H5	120.0	C12—C13—H13	119.7
C4—C5—H5	120.0	C14—C13—H13	119.7
C5—C6—C7	116.52 (15)	C13—C14—C9	120.05 (17)
C5—C6—H6	121.7	C13—C14—H14	120.0
C7—C6—H6	121.7	C9—C14—H14	120.0
O1—C7—C6	125.39 (14)	S1—C15—H15A	109.5
O1—C7—C2	110.46 (14)	S1—C15—H15B	109.5
C6—C7—C2	124.14 (16)	H15A—C15—H15B	109.5
C1—C8—O1	110.58 (14)	S1—C15—H15C	109.5
C1—C8—C9	135.33 (15)	H15A—C15—H15C	109.5
O1—C8—C9	114.07 (13)	H15B—C15—H15C	109.5
C10—C9—C14	116.90 (16)		
			
C7—C2—C3—C4	0.5 (2)	C9—C8—C1—C2	−177.68 (19)
C1—C2—C3—C4	−178.32 (19)	O1—C8—C1—S1	−170.46 (12)
C2—C3—C4—C5	−1.0 (3)	C9—C8—C1—S1	11.4 (3)
C2—C3—C4—Br1	178.97 (12)	C7—C2—C1—C8	−0.58 (19)
C3—C4—C5—C6	1.1 (3)	C3—C2—C1—C8	178.35 (19)
Br1—C4—C5—C6	−178.93 (14)	C7—C2—C1—S1	170.58 (13)
C4—C5—C6—C7	−0.5 (3)	C3—C2—C1—S1	−10.5 (3)
C8—O1—C7—C6	−178.84 (18)	O2—S1—C1—C8	137.85 (16)
C8—O1—C7—C2	−0.24 (19)	C15—S1—C1—C8	−113.90 (17)
C5—C6—C7—O1	178.44 (17)	O2—S1—C1—C2	−31.49 (17)
C5—C6—C7—C2	0.0 (3)	C15—S1—C1—C2	76.75 (16)
C3—C2—C7—O1	−178.64 (15)	C14—C9—C10—F1	−176.38 (15)
C1—C2—C7—O1	0.50 (19)	C8—C9—C10—F1	−0.1 (3)
C3—C2—C7—C6	0.0 (3)	C14—C9—C10—C11	1.8 (3)
C1—C2—C7—C6	179.13 (17)	C8—C9—C10—C11	178.11 (17)
C7—O1—C8—C1	−0.15 (19)	F1—C10—C11—C12	177.61 (17)
C7—O1—C8—C9	178.42 (14)	C9—C10—C11—C12	−0.6 (3)
C1—C8—C9—C10	32.8 (3)	C10—C11—C12—C13	−0.9 (3)
O1—C8—C9—C10	−145.32 (16)	C11—C12—C13—C14	1.0 (3)
C1—C8—C9—C14	−151.1 (2)	C12—C13—C14—C9	0.2 (3)
O1—C8—C9—C14	30.8 (2)	C10—C9—C14—C13	−1.6 (3)
O1—C8—C1—C2	0.46 (19)	C8—C9—C14—C13	−177.98 (16)

Symmetry code: (i) −*x*+1, −*y*+1, −*z*.

### Hydrogen-bond geometry (Å, º)

**Table:**

*D*—H···*A*	*D*—H	H···*A*	*D*···*A*	*D*—H···*A*
C6—H6···O1^ii^	0.95	2.52	3.4633 (19)	173
C14—H14···O2^iii^	0.95	2.44	3.365 (2)	164

Symmetry codes: (ii) −*x*, −*y*+1, −*z*+1; (iii) −*x*+1, −*y*+1, −*z*+1.
